# Supplementary material for: The human immunoglobulin heavy chain constant gene locus is enriched for large complex structural variants and coding polymorphisms that vary in frequency among human populations
Source: bioRxiv. 2025 Feb 12:2025.02.12.634878. Preprint. [Version 1] doi: 10.1101/2025.02.12.634878 (PMC11844466; doi:10.1101/2025.02.12.634878)
Supplement: Supplementary file 1 [file media-1.pdf]

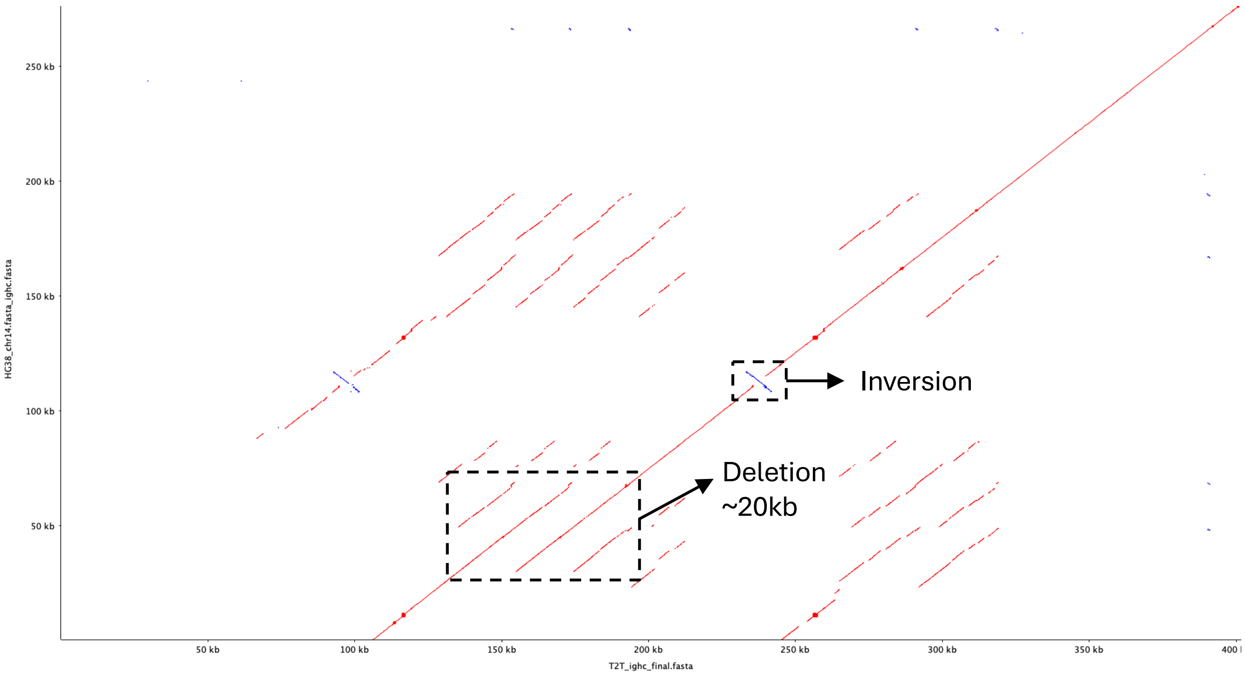

Figure S1. A dot plot based comparison between the IGHC loci obtained from GRCh38 (X axis) and T2TCHM13v2.0 (Y axis) reference assembly.

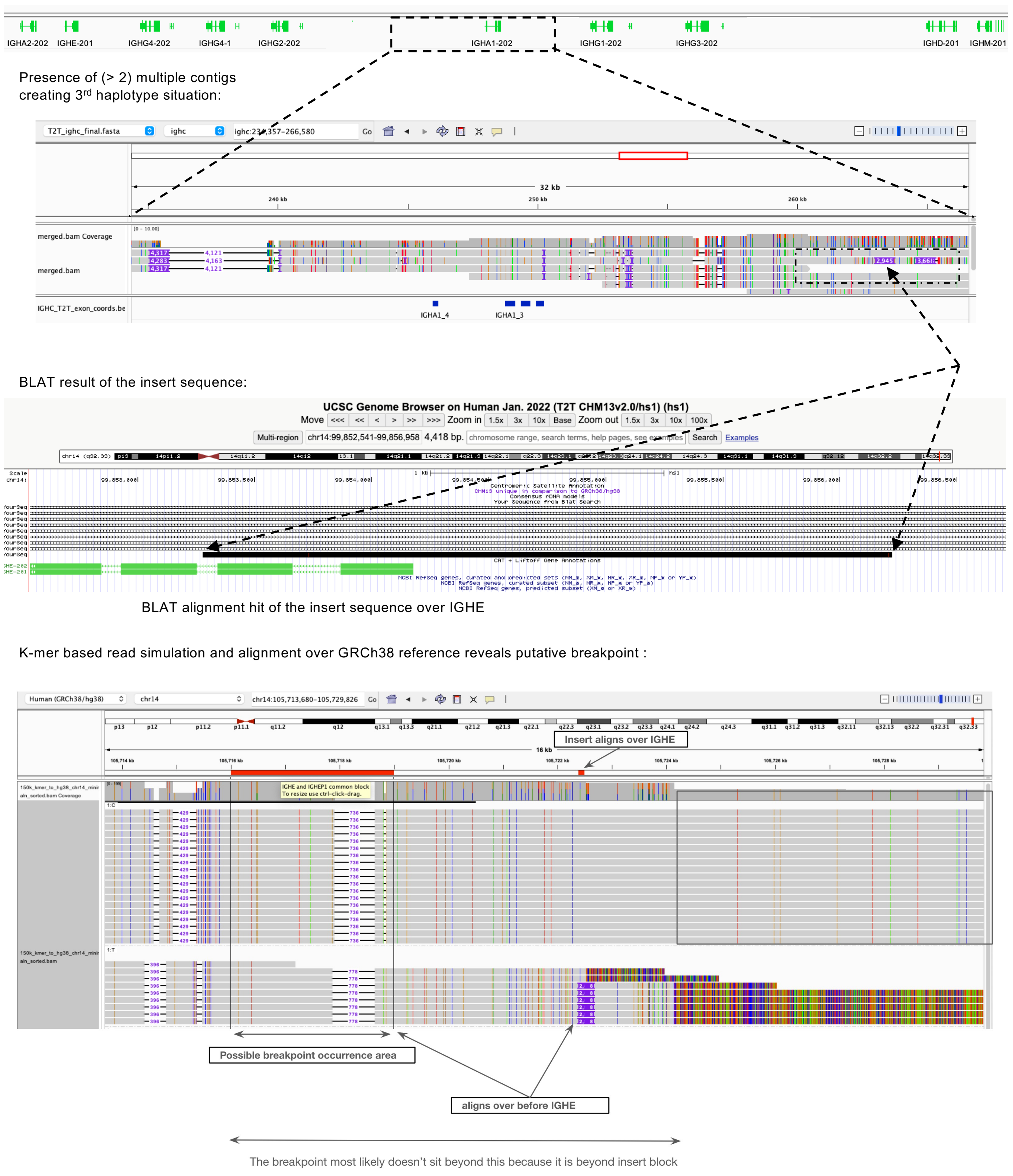

Figure S2. Identification of break points in large segmental duplication block observed in the Hifiasm de-novo assembly contigs.
The presence of a large segmental duplication was confirmed through the identification of a third haplotype within the alignment. Subsequently, the mapping locations of the inserted sequences (~2–3 kb) were determined using the UCSC Genome Browser BLAT toolkit. The BLAT results revealed alignment over IGHE rather than the pseudogene (EP), providing initial insights into the potential breakpoints. Furthermore, a read simulation approach was employed using the resolved duplicated haplotype to generate 150 kb-sized k-mers as reads. These simulated reads were then aligned to the reference genome to gain a clearer visual representation of the putative breakpoint.

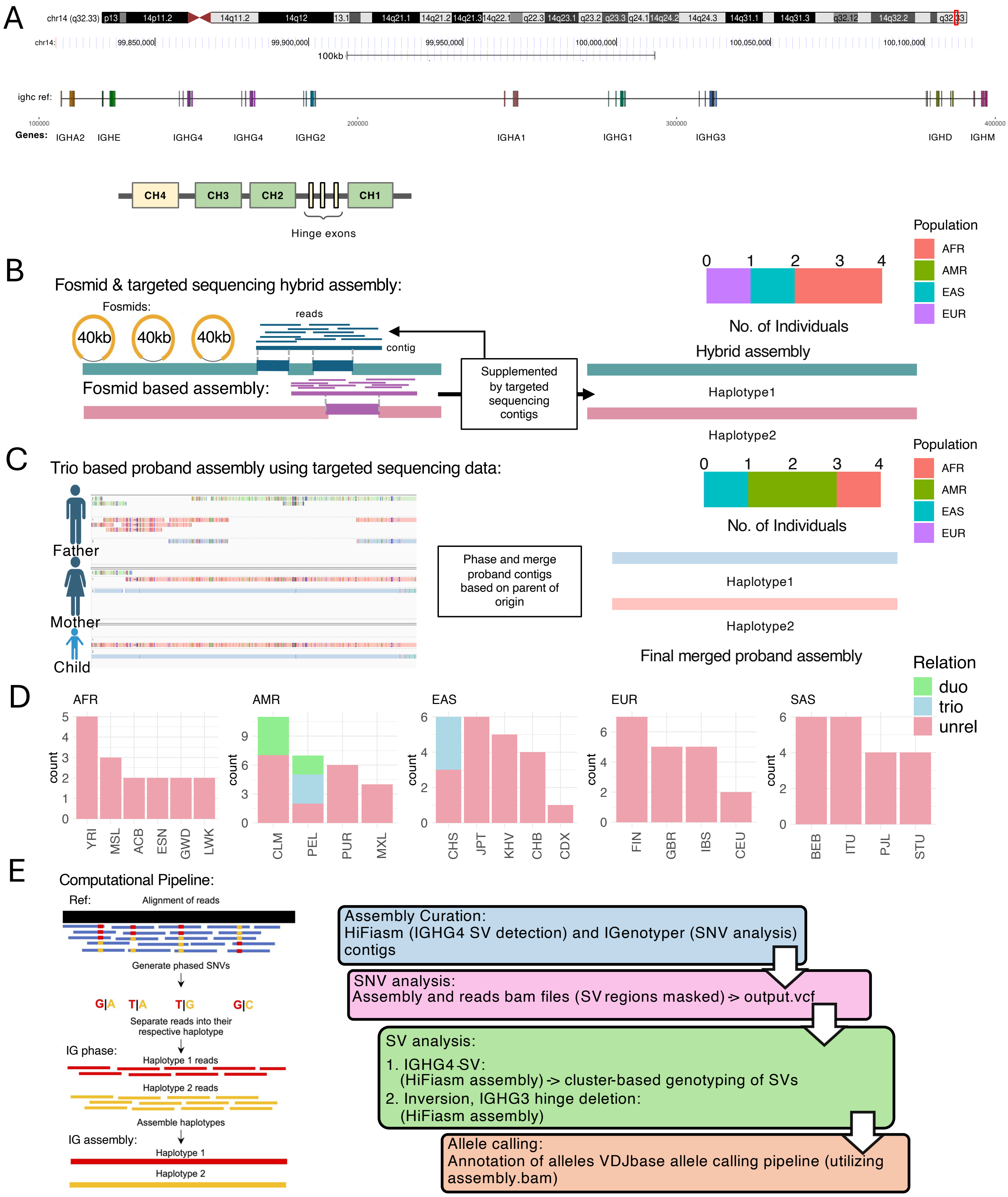

Figure S3. A comprehensive overview of methods ad samples used for analyzing IGHC genomic diversity (A) schematic map of the IGHC locus and gene structure. **(B-C)** Schematic representation of the selected ground truth assemblies - fosmid, long-read targeted capture data was used to produce highly accurate hybrid assemblies; targeted long read sequence from both parents and proband (trio) was used to produce the second set of highly vetted assemblies. **(D)** A breakdown of relation and ancestral information for the 105 individuals used in the study. **(E)** The schematic representation of high-throughput computational pipeline used for analyzing IGHC locus.

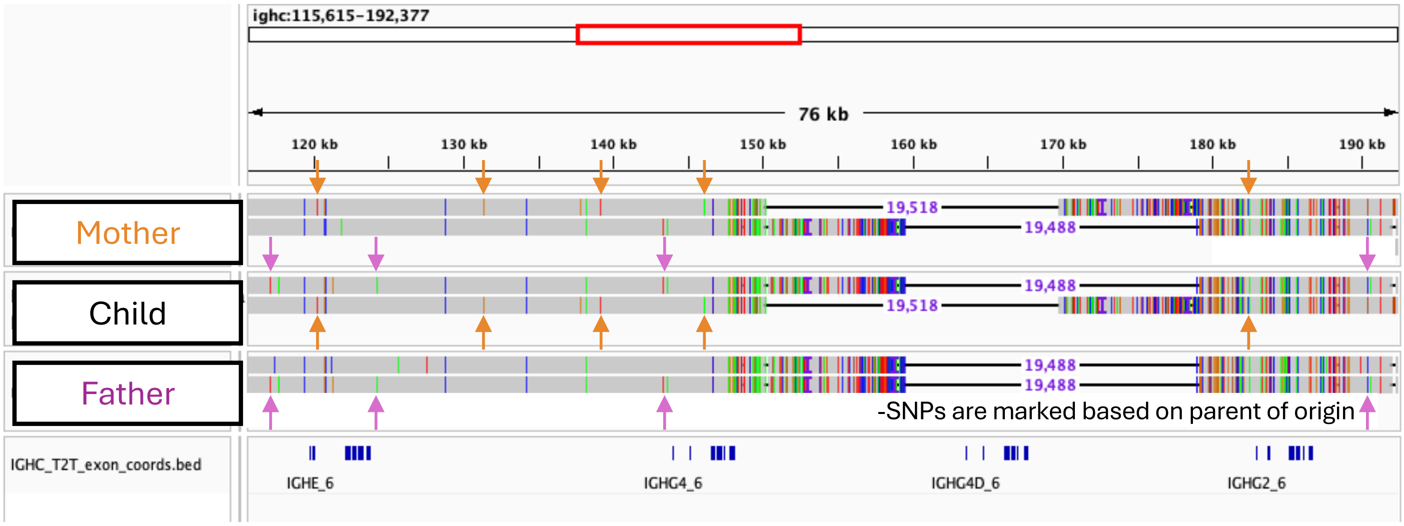

Observing child assembly alongside parental assemblies :

- Orange arrow marked SNPs are maternal haplotype guide within the proband contig
- Purple arrow marked SNPs are paternal haplotype guide within the proband contig
- Parallel observation of paternal and maternal contigs for ensuring accurately phased proband contigs

Figure S4. A schematic representation for manually ensuring correct phasing in trio-based proband assemblies.

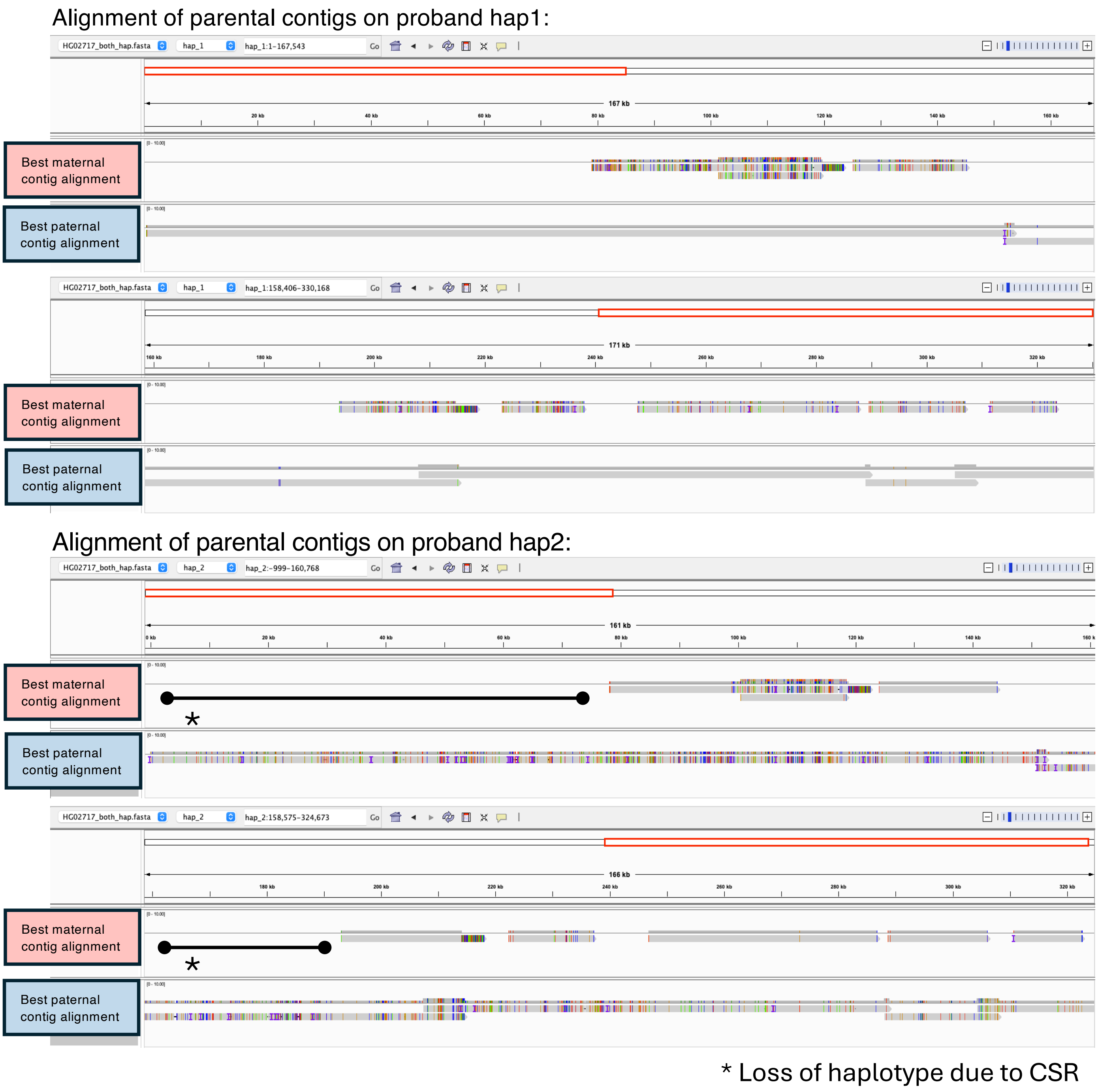

Figure S5. Representation of correct phasing in scenarios harboring loss of haplotype
Illustrating here is a Haplotype loss as observed in the mother's assembly (HG02716); likely due to a class-switch event. However, complete paternal assembly (HG02715) and partial maternal assembly data proved adequate for reconstructing the proband assembly (HG02717).

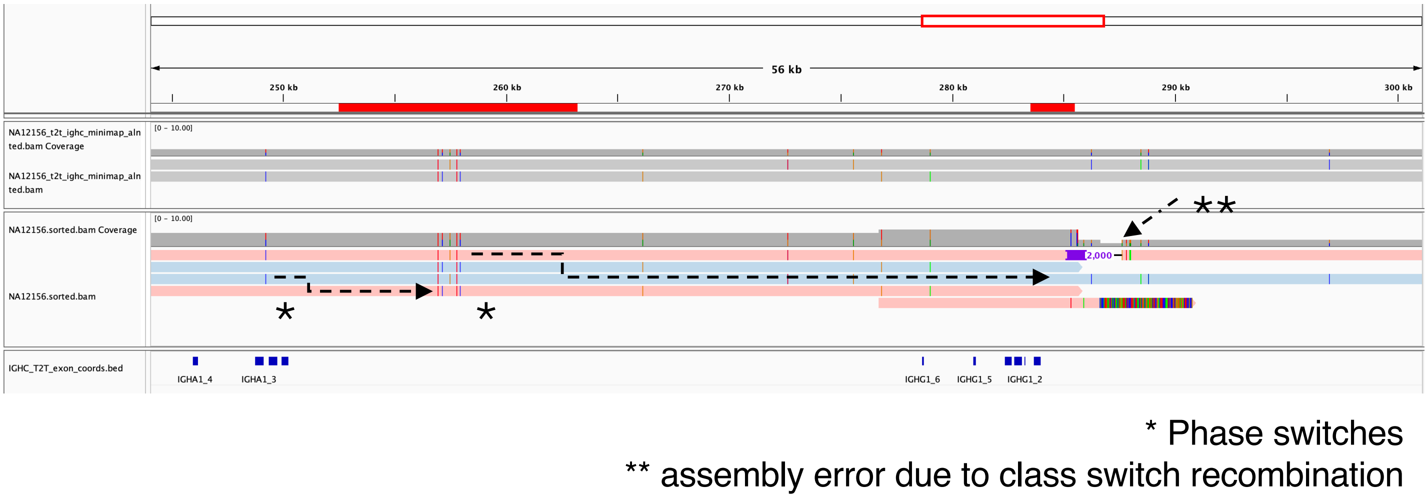

Figure S6. An example representation of phase switch errors and mis-assembly caused by class-switch recombination.
The dotted arrows (marked with *) connecting haplotypes indicate the correct phasing over the shown haplotypes. The contiguous assembly contig over the region (marked with **), with an exact haplotype representing contig with right soft-clipping, points to the presence of a class-switch event (DNA rearrangement placing VDJ sequences in position) after IGHG1, which results in mis-assembly in the primary assembly contigs.

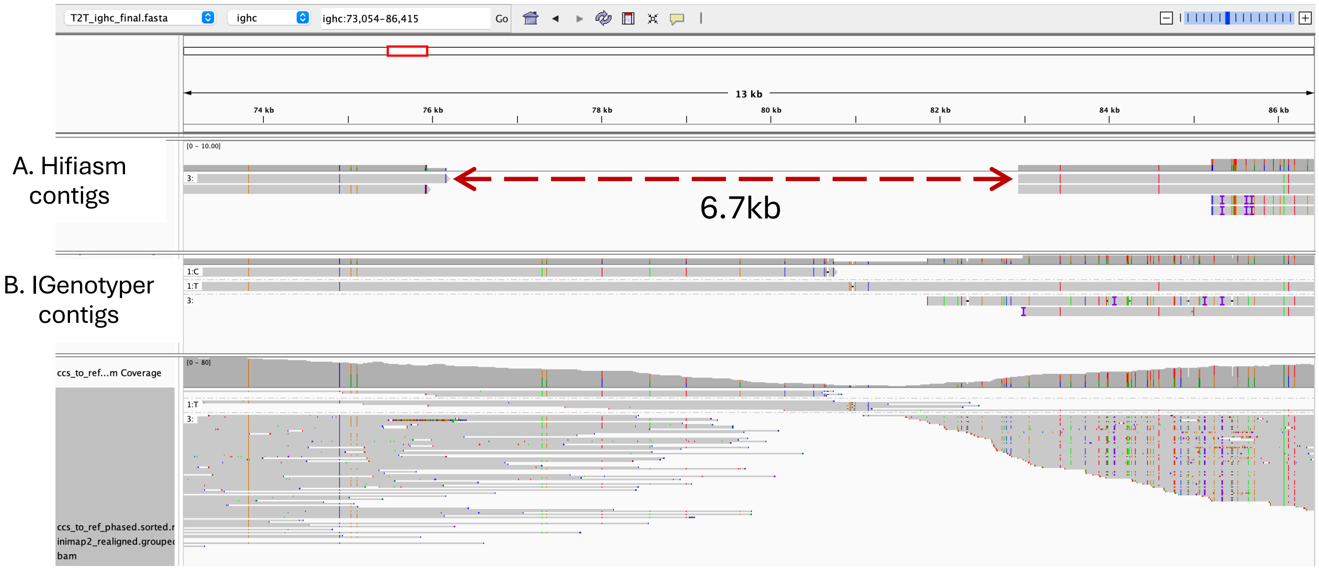

Figure S7. An example of a comparison for assembly contig generation between IGenotyper and Hifiasm. IGenotyper extends/produces contig over lower depth, whereas Hifiasm skips the extension of the contig at such regions.

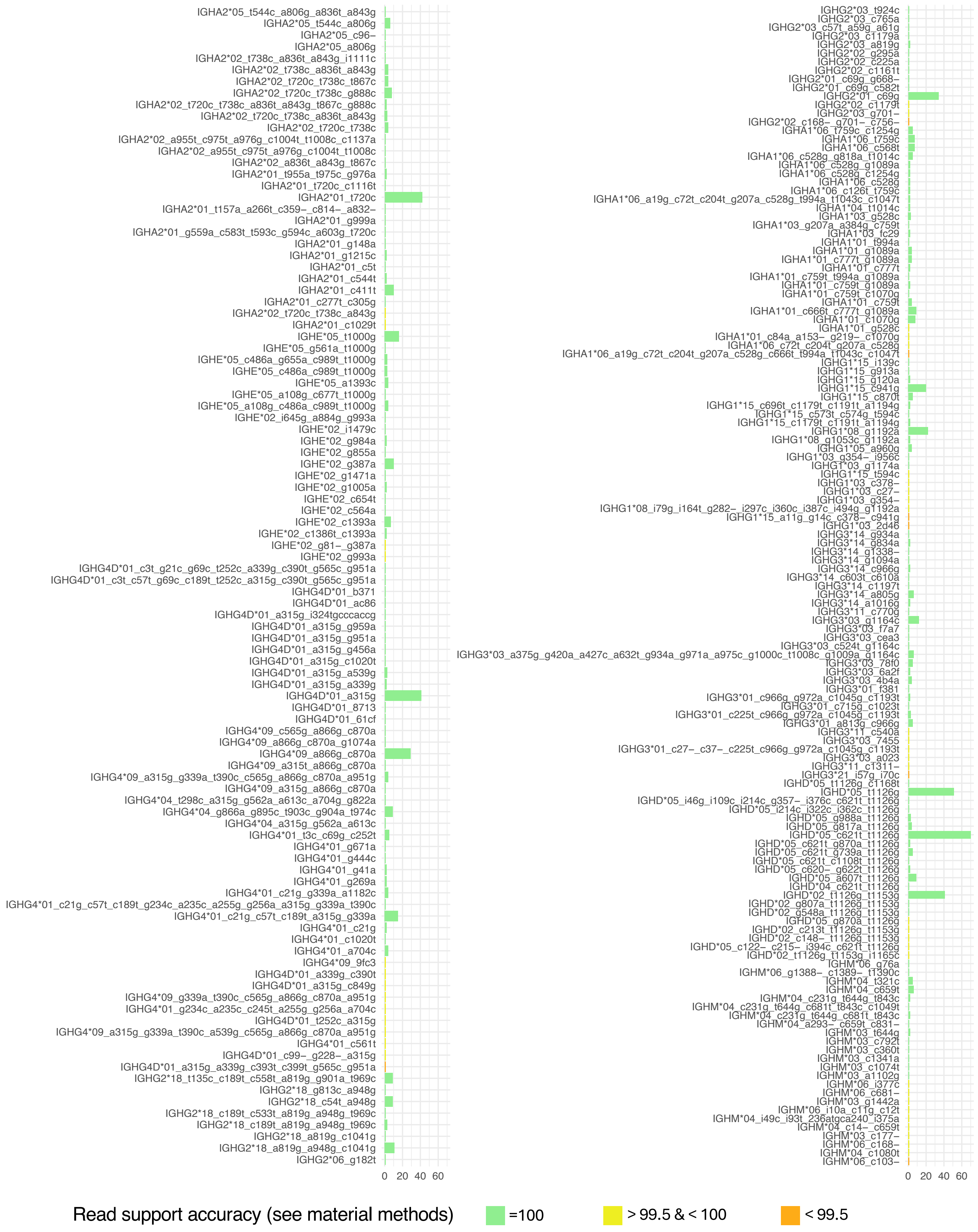

Figure S8. Bar plot for frequency distribution of novel alleles observed in this study
Here “X” axis indicates no. of individuals observed with the novel allele and the color of the horizontal bar indicates read based accuracy support for the allele

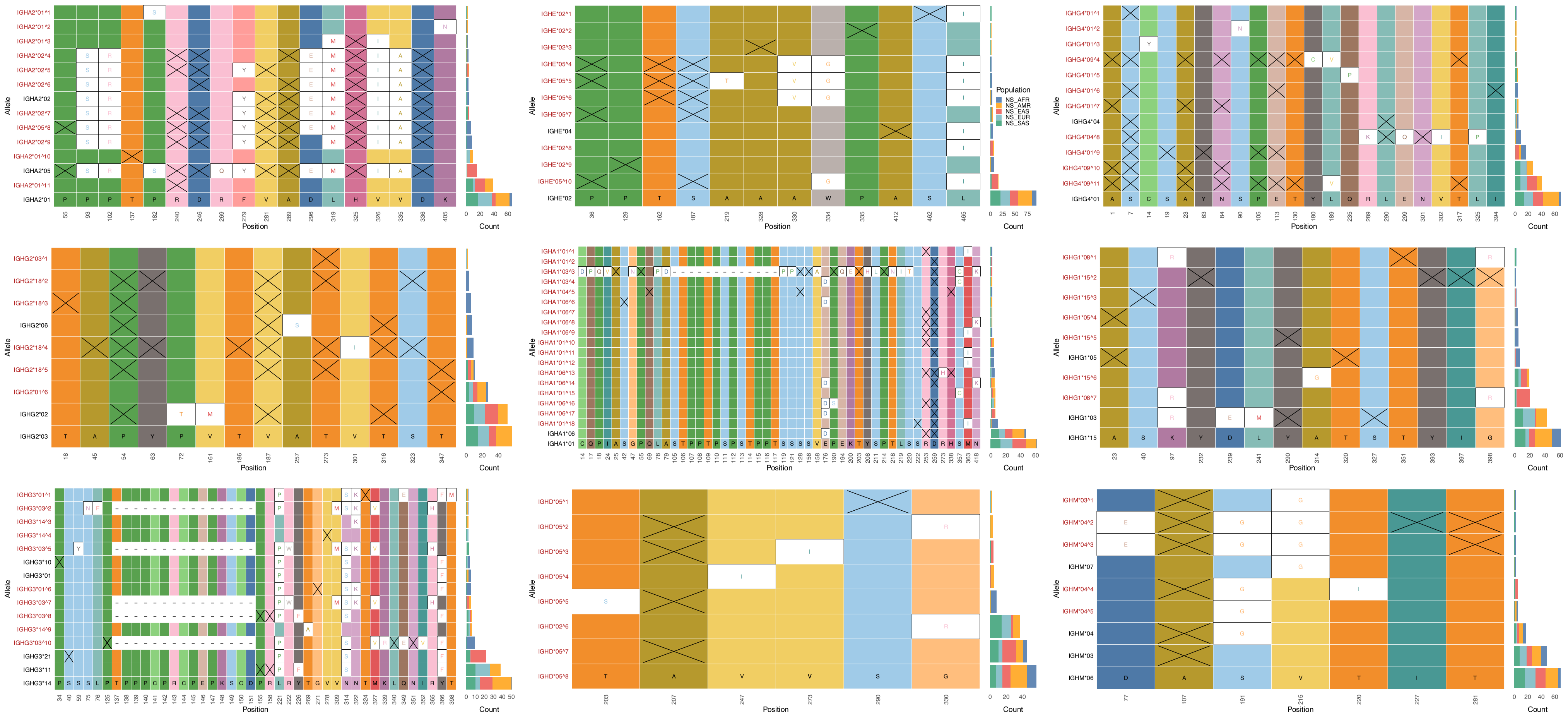

Figure S9. SNV analysis reporting synonymous, non-synonymous changes compared to most frequent allelic variety as observed in all IGHC alleles (present in > 1 individual)

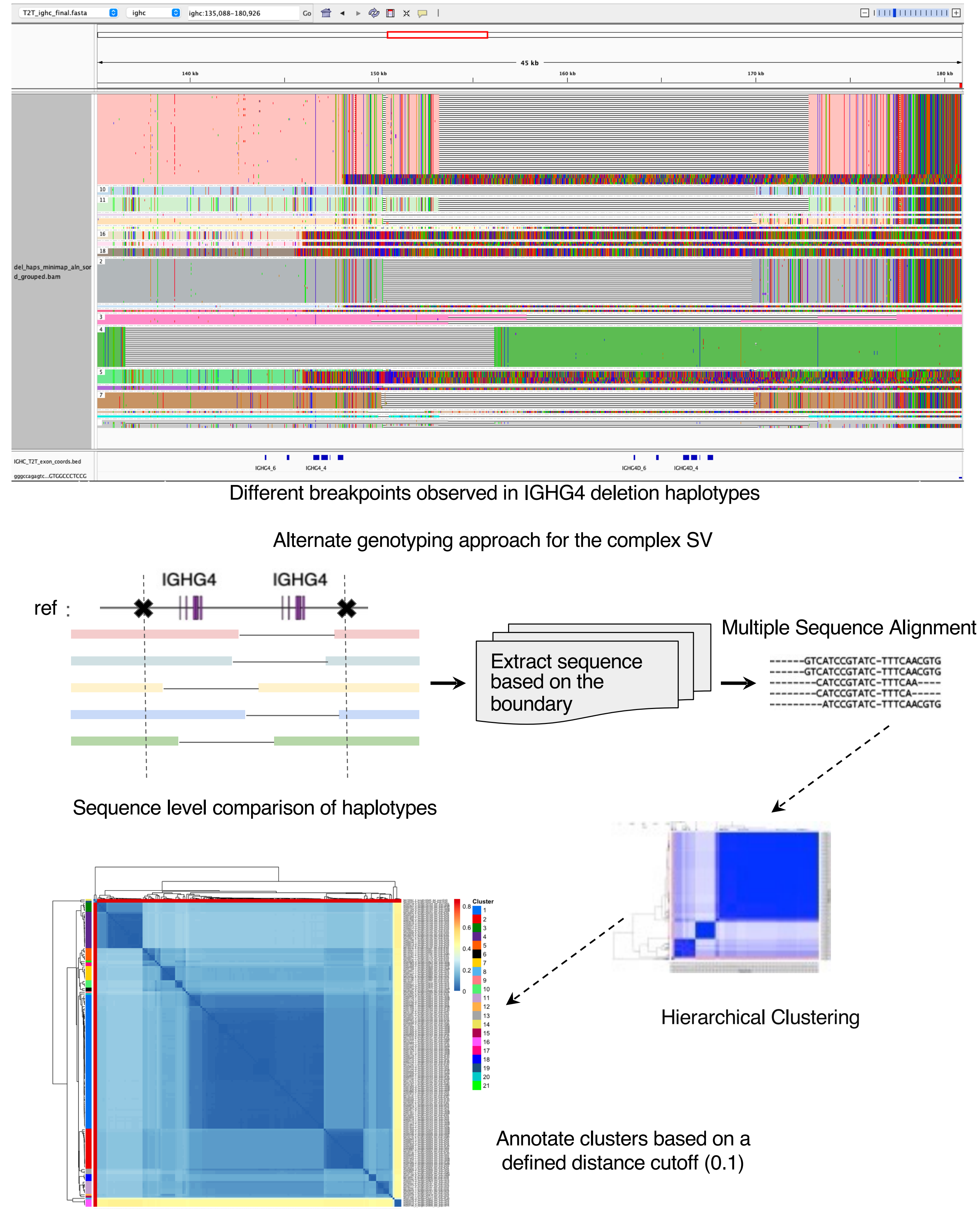

Figure S10. Schematic representation of Genotyping approach for the complex IGHG4 deletion SVs.

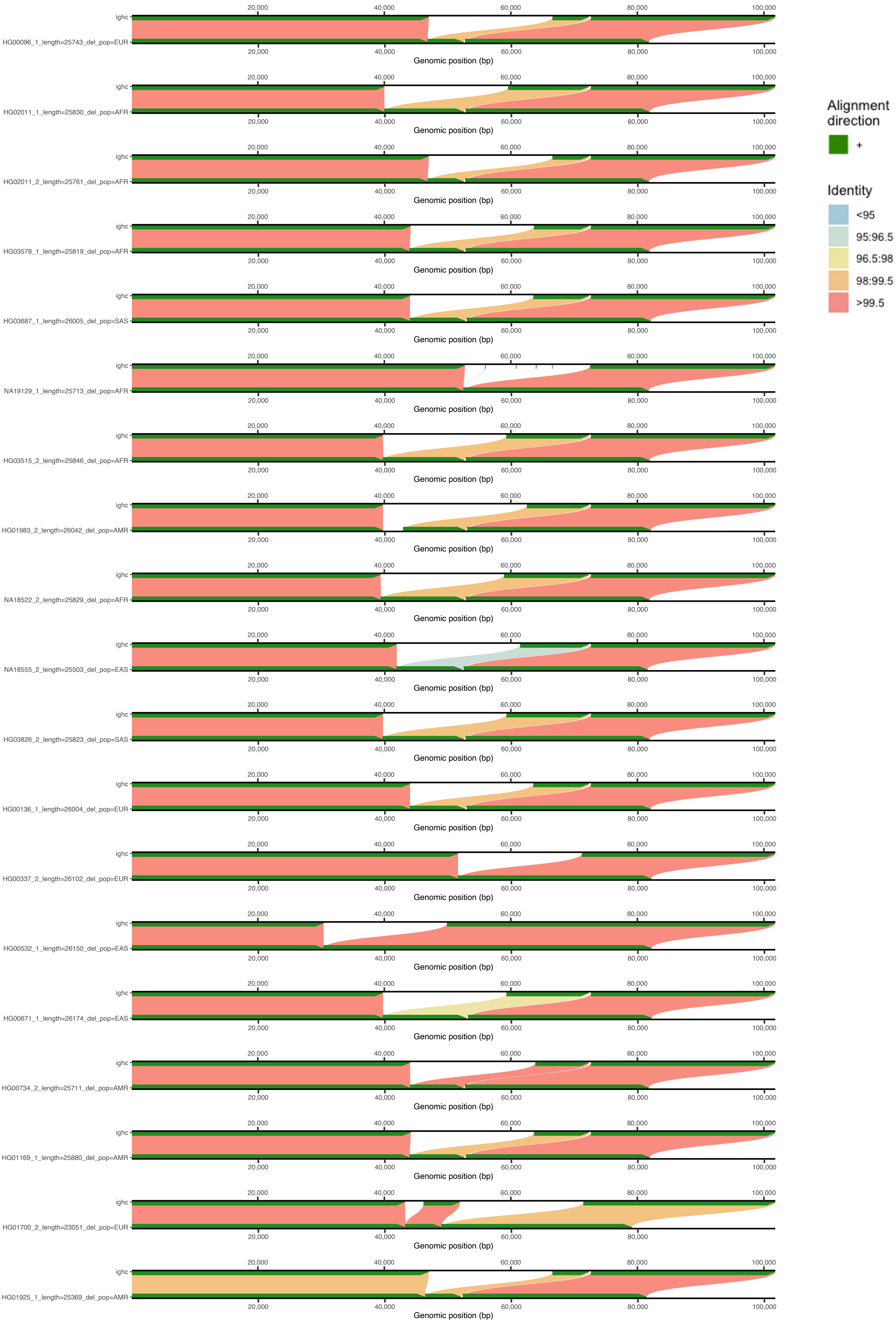

Figure S10. Schematic representation of Genotyping approach for the complex IGHG4 deletion SVs (cont.)
Here is a representation of the haplotype clusters obtained based on distance-based genotyping, showing how the deletion breakpoints compare with each other in terms of alignment over the specific IGHC region, using the CHM13v2.0 reference and analyzed with SVbyEye (Porubsky, David et al).

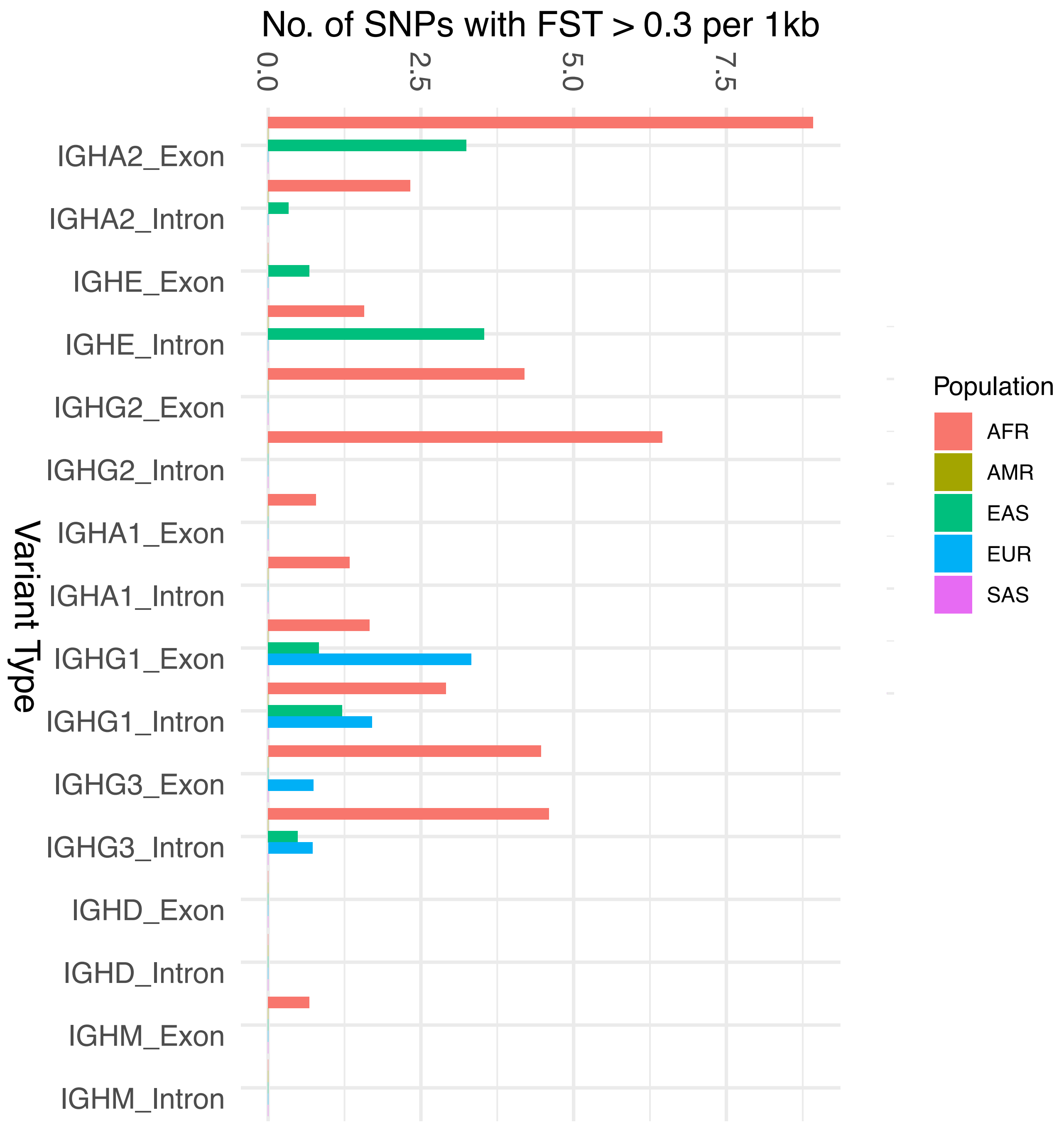

Figure S11. Bar plot showing distribution of SNVs in the IGHC locus with high F_{ST} values across different super-population.

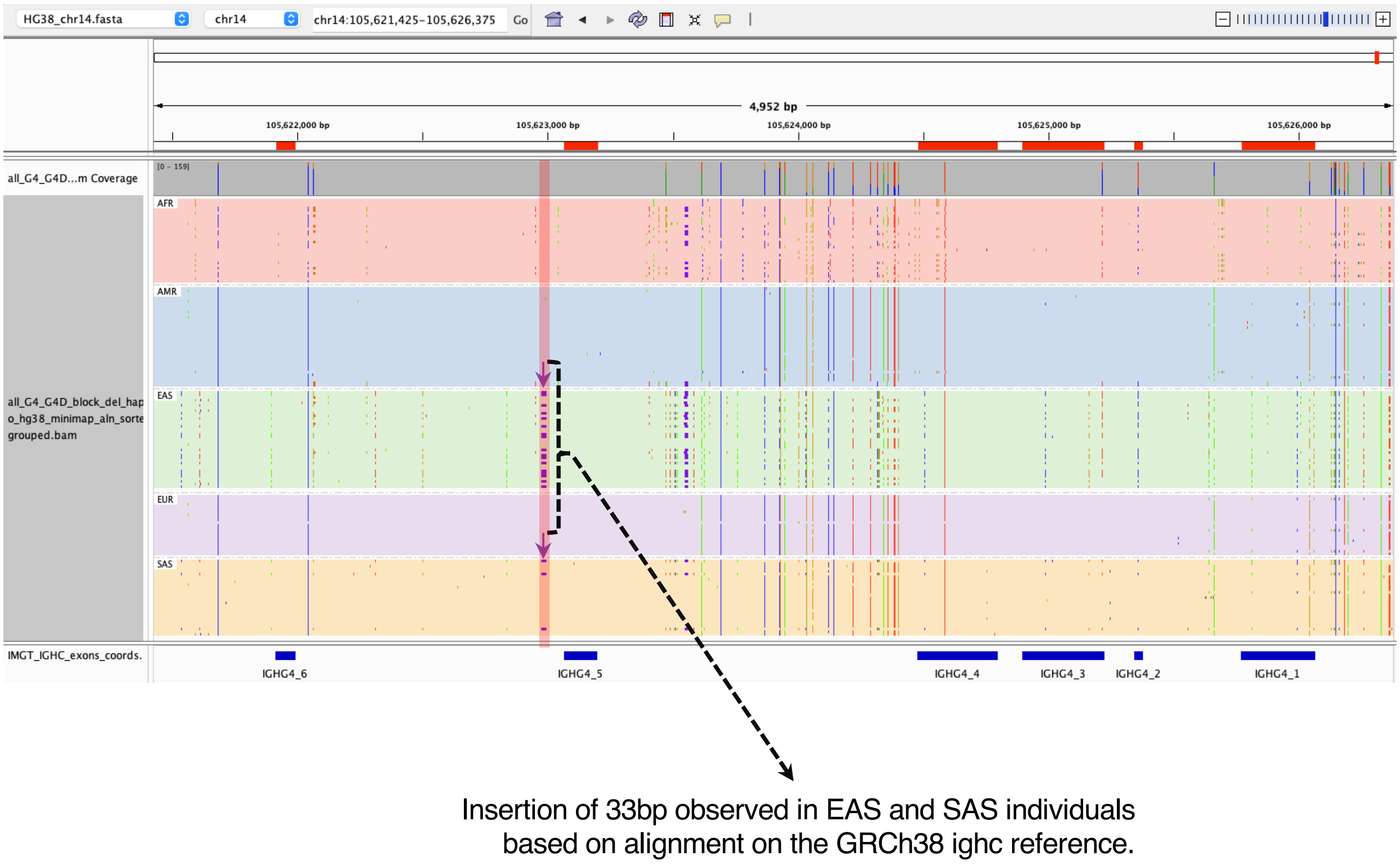

Figure S12. Observation of ancestry defined evolutionary signature in the IGHC locus.
The ancestry-partitioned alignment of haplotypes over the IGHC locus in the GRCh38 reference suggests the presence of a 33bp nucleotide insertion signature in the intronic region of IGHG4, which was previously reported as evidence of Neanderthal introgression by Yan et al. (2021). Our study aligns with their findings and further supports the higher ancestral bias for this insertion among haplotypes from the EAS and SAS super-populations.

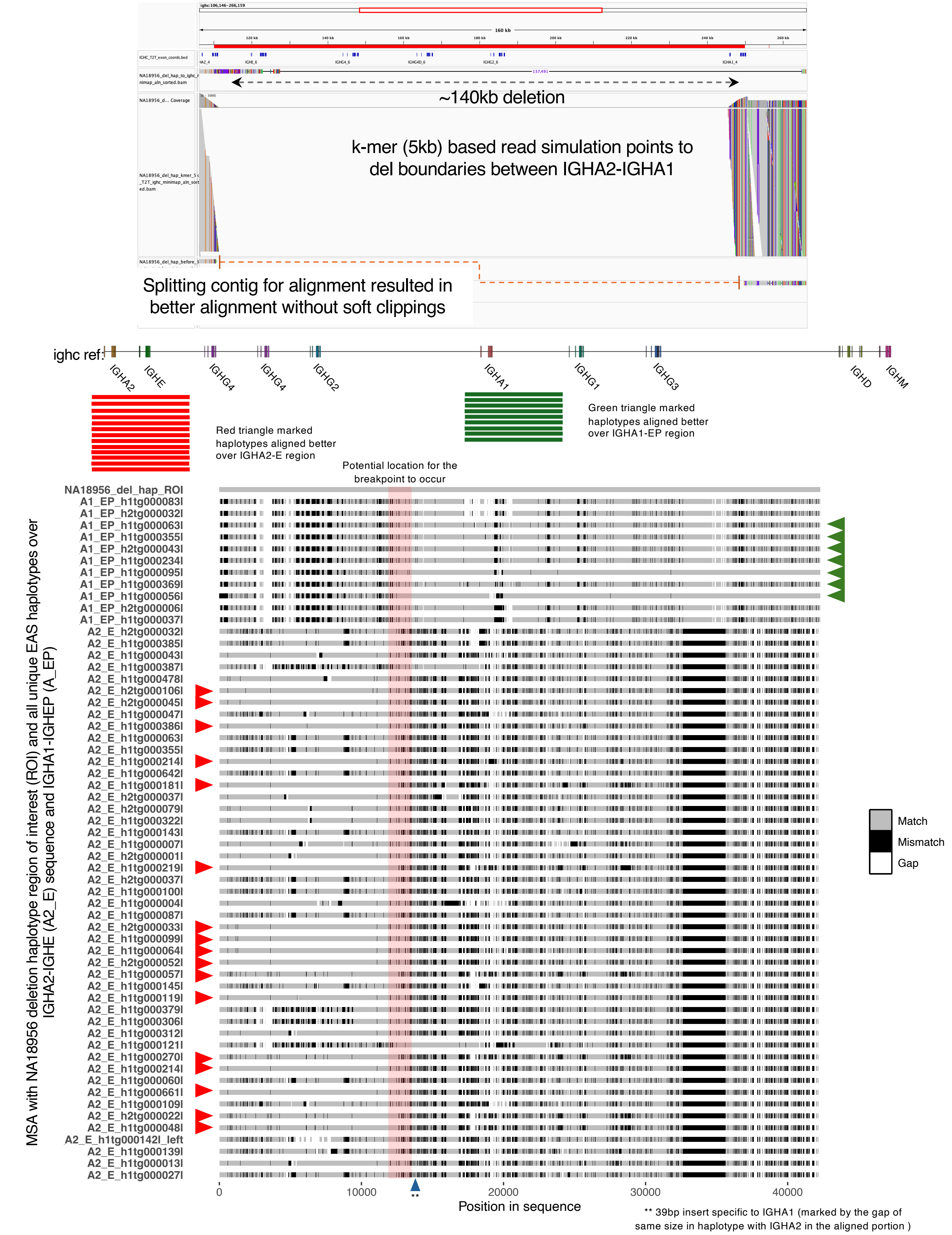

Figure S13. Schematic for potential breakpoint estimation in the multigene deletion haplotype (NA18956)

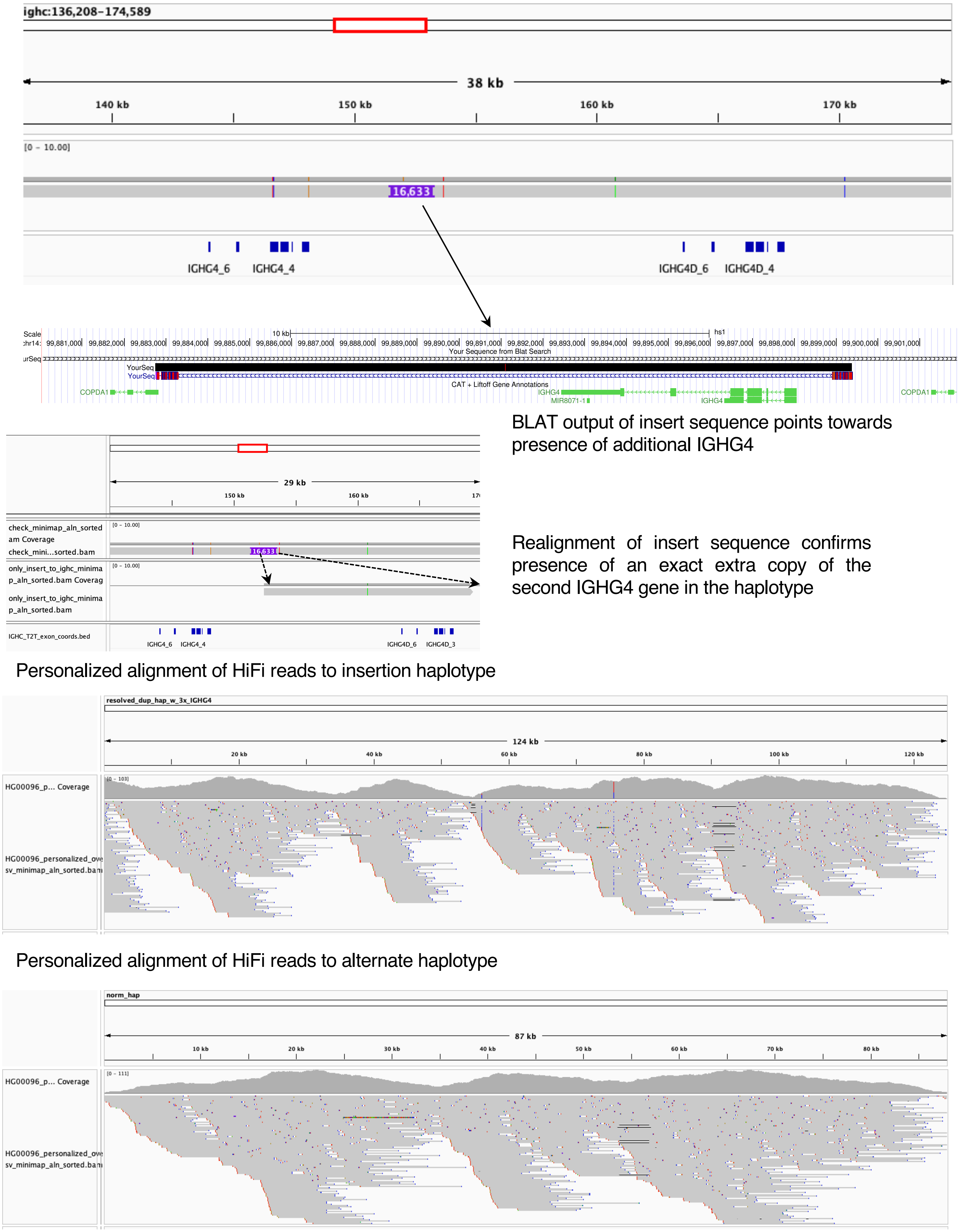

Figure S14. Mapping of 3CNV IGHG4 haplotype over IGHC reference from T2TCHM13v2.0.
The EUR haplotype contains two copies of IGHG4, identical to the reference, along with a ~16kb insert between the genes. Subsequent alignment of the insert sequence reveals the presence of a third copy.

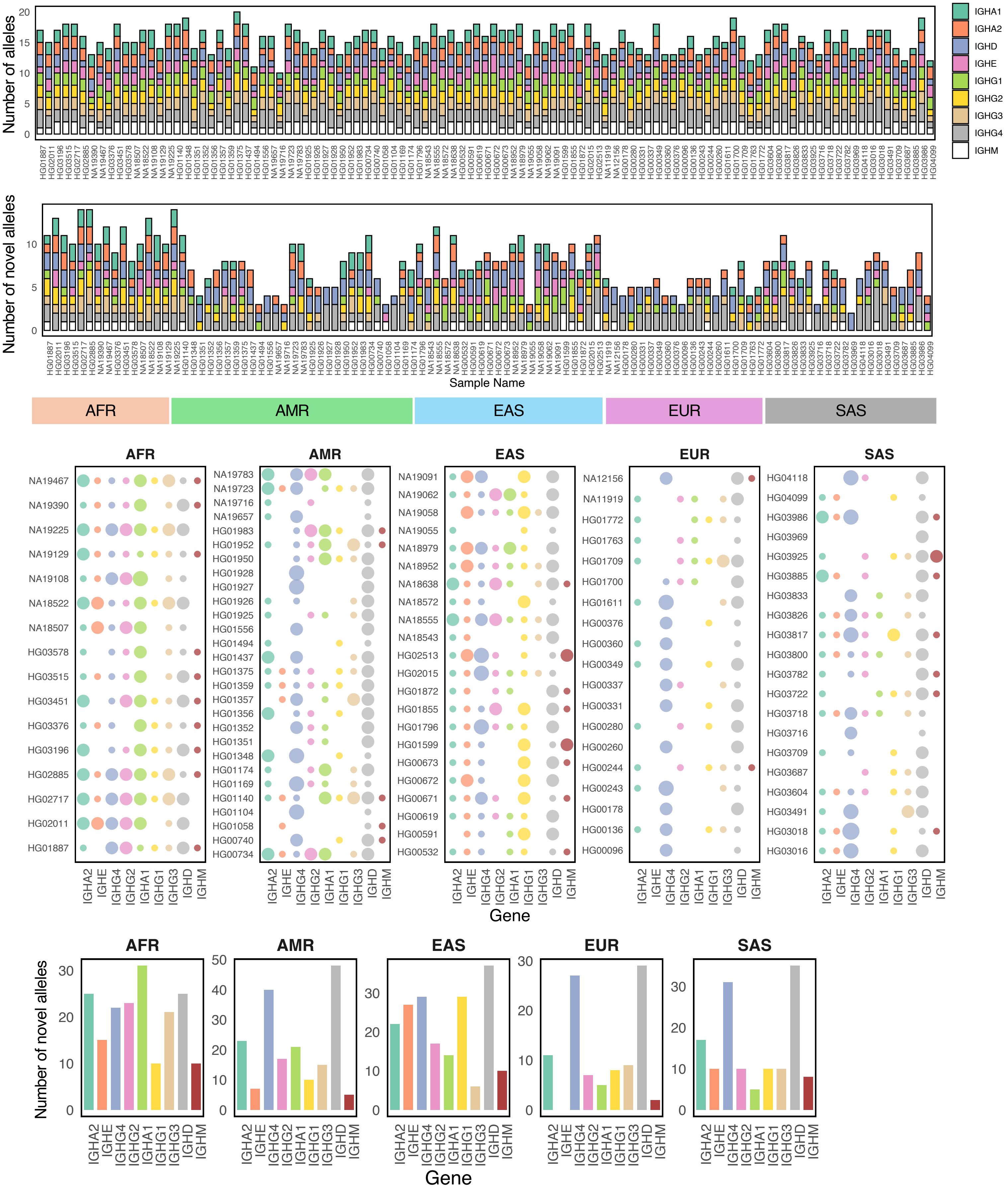

Figure S15. Distribution of novel alleles over IGHC genes in individuals across diverse super-populations.
